# A functional neuron maturation device provides convenient application on microelectrode array for neural network measurement

**DOI:** 10.1186/s40824-022-00324-z

**Published:** 2022-12-20

**Authors:** Xiaobo Han, Naoki Matsuda, Yuto Ishibashi, Aoi Odawara, Sayuri Takahashi, Norie Tooi, Koshi Kinoshita, Ikuro Suzuki

**Affiliations:** 1 Department of Electronics, Graduate School of Engineering, Tohoku Institute of Technology, 35-1 Yagiyama Kasumicho, Taihaku-Ku, Sendai, Miyagi 982-8577 Japan; 2 Stem Cell & Device Laboratory, Inc. (SCAD), OFFICE-ONE Shijo Karasuma 11F, 480, Niwatoriboko-Cho, Shimogyo-Ku, Kyoto, 600-8491 Japan

**Keywords:** SCAD device, Microelectrodes array, Convenient application, Human induced pluripotent stem cells, Peripheral neurons, Neural network function, In vitro to in vivo extrapolation

## Abstract

**Background:**

Microelectrode array (MEA) systems are valuable for in vitro assessment of neurotoxicity and drug efficiency. However, several difficulties such as protracted functional maturation and high experimental costs hinder the use of MEA analysis requiring human induced pluripotent stem cells (hiPSCs). Neural network functional parameters are also needed for in vitro to in vivo extrapolation.

**Methods:**

In the present study, we produced a cost effective nanofiber culture platform, the SCAD device, for long-term culture of hiPSC-derived neurons and primary peripheral neurons. The notable advantage of SCAD device is convenient application on multiple MEA systems for neuron functional analysis.

**Results:**

We showed that the SCAD device could promote functional maturation of cultured hiPSC-derived neurons, and neurons responded appropriately to convulsant agents. Furthermore, we successfully analyzed parameters for in vitro to in vivo extrapolation, i.e., low-frequency components and synaptic propagation velocity of the signal, potentially reflecting neural network functions from neurons cultured on SCAD device. Finally, we measured the axonal conduction velocity of peripheral neurons. Conclusions: Neurons cultured on SCAD devices might constitute a reliable in vitro platform to investigate neuron functions, drug efficacy and toxicity, and neuropathological mechanisms by MEA.

**Supplementary Information:**

The online version contains supplementary material available at 10.1186/s40824-022-00324-z.

## Background

Traditional methods for drug screening and neurotoxicity testing largely rely on cellular and animal models [1] . Recently, there has been increasing use of human neurons and neural networks generated from human induced pluripotent stem cells (hiPSCs) and embryonic stem cells because of their ability to differentiate into specific human cell types [2–5] . These neurons are used in assays to evaluate neuronal differentiation, neurite growth, synaptogenesis, neuronal network formation, and substance toxicity. [6, 7] Numerous screening of drugs relevant to dysfunctions of the mammalian central nervous system using hiPSC-derived cells focused on electrophysiological functions. Indeed, the electrical activity of neural network is critical for the generation and transmission of information conduction under physiological conditions [8–11] . Microelectrode array (MEA) systems are valuable for the noninvasive, real-time, and multipoint measurement of the electrophysiological activity of cultured mammalian neuronal networks and brain slices [12–17] . Previous work showed the effectiveness of MEAs for measuring the response to drugs in hiPSC-derived neurons [18–24] .

However, the development of in vitro MEA technologies using hiPSC-derived neurons still face several difficulties to be improved. Firstly, the in vitro maturation of hiPSC-derived neurons in culture to produce cells presenting the electrophysiological and pharmacological characteristics of human neuronal networks found in vivo lasts a relatively long time (6 weeks or more) [25] . Our and other groups showed that the co-culture with astrocytes effectively promotes neuron maturation [21, 22, 26, 27]. A culture system should be welcomed for a faster maturation with neurons alone. Secondly, the culture of neurons for MEAs requires using an appropriate extracellular matrix to enhance cell adhesion and prevent cell aggregation [21, 22] . This might be challenging as different types of hiPSCs require specific materials on the surface of different MEA plates for seeding. Thirdly, researchers recommended using new MEA probes for each experiment, and MEA probes are quite expensive. Thus, a successful data acquisition in MEA assays might be costly and time-consuming. Finally, an in vitro to in vivo extrapolation (IVIVE) is required in MEA using hiPSC-derived neurons [28–31]. Traditional MEA measurements are preferred to analyze spikes with frequency components of 1 kHz or more and to investigate pathological mechanisms or drug efficacy [32]. New approach methods are required to investigate in vitro the activity of neural networks and predict in vivo activities.

Recently, numerous models with cells cultured on scaffolding biomaterials providing an adequate surface for cell adhesion and enabling efficient cell proliferation, differentiation, and organization into a mature and functional engineered tissue have been developed [33–35]. For neurons, scaffolds made of processed fibers of synthetic polymers contribute to the parallel axon guidance by mimicking the native tissue environment [36–38]. However, most systems are not suitable for long-term in vitro culture. Moreover, the electrophysiological properties of neurons cultured on these polymers have not been evaluated. In the present study, we produced a novel cell culture platform named SCAD (Stem Cell & Device) device and consisting of a frame of aligned electrospun polystyrene (ESPS) fibers. The SCAD device provides a quickly and convenient set up on multiple MEA probes just before measurement. For cell culture, we showed that the SCAD device allowed stable long-term culture (over 100 days), and promoted a faster maturation of hiPSC-derived neurons compared with that of neurons cultured directly on MEA probes. The SCAD device also enabled in vitro neural network analyses. In particular, we successfully analyzed the low-frequency components of local potential fields, which reflect neural network functions, and the action potential propagations between neural networks using the SCAD device and MEAs.

## Methods

### SCAD device fabrication

A solution of 25% polystyrene (Sigma-Aldrich, 182,435) in N,N-Dimethylformamide (FUJIFILM Wako, 047–29,191) was prepared and vigorously mixed for 30 min. Then, the mixed solution was vertically rotated at low speed (1 rotation per 25 s) for 10 h. The resultant solution was transferred into a 5-ml plastic syringe (HJ4050-LL, OSAKA CHEMICAL Co., Ltd.) with a 25-gauge needle (490,732, BSA Sakurai) and loaded into the electrospinning apparatus (NANON-04, MECC CO., LTD). Electrospinning of the solution was performed at ambient temperature, with an applied voltage of 9–10 kV DC, injection rate of 1.7 ml/h, tip-to-collector distance of 130 mm, and rotational speed of 2,000 rpm for 13 min once or 6.5 min twice, and fibers were collected throughout. The fibers were collected onto a commercial A4 size paper (0001-PRKA4-BK-01, Etranger di Costarica) and pasted with glue (KE-45-T, Shin-Etsu Chemical Co., Ltd.) to stainless-steel washer with a polycarbonate frame.

### SCAD device preparation and cell culture

Before cell seeding, the SCAD device was subjected to 4 min of plasma treatment (PC-40 T, STREX Inc.) followed by 1 min of UV irradiation. Then, the device was coated overnight at 37 °C with 0.02% poly-L-ornithine (P4957, Sigma-Aldrich). After washing with phosphate-buffered saline (PBS), the device was coated with 2.5 µg/mL laminin 511 (381–07,363, Wako) for 2 h at 37 °C.

Cryopreserved hiPSC-derived cortical neurons (XCL-1 Neurons, XCell Science) were thawed and suspended in Neuron Medium (XCS-NM-001-M100-1P, XCell Science). For dispersed culture, 5.4 × 10^4^ cells (6.0 × 10^5^ cells/cm^2^) in 20 µL Neuron Medium were seeded on a treated SCAD device. Two hours later, devices with cells were transferred into a well of a 24-well plate filled with 1 mL Neuron Medium. After 1 week, the medium was replaced with 1 mL of BrainPhys Neuronal Medium containing SM1 neuronal supplement (ST-05792, STEMCELL technologies), and 20% Astrocyte conditioned medium (1811-sf, ScienCell Reserch Labratories). Afterward, half the volume of the medium was replaced twice per week.

DRG neurons were harvested and cultured as described previously [57] . Briefly, DRG neurons were collected from 10 weeks old male Wistar Rats. The ethical approval for this study was obtained from Tohoku Institute of Technology Animal Care and User Committee. Firstly, rats were asphyxiated with isoflurane and then decapitated. DRGs were harvested from the vertebral column, and the sensory neurons were dissociated by mechanical agitation after incubation for 2 h with collagenase type III (CLS3, Worthington) at 37 °C. Then, cells were washed with Hank’s balanced salt solution and further dissociated with trypsin type I (T8003, Sigma-Aldrich). After cell counting, approximately 5 × 10^4^ cells (6.0 × 10^5^ cells/cm^2^) in 20 µL BrainPhys Neuron Medium were seeded on a treated SCAD device. One hour later, devices with cells were transferred into a well of a 24-well plate filled with 1 mL BrainPhys Neuron Medium. The next day, the medium was replaced with 1 mL of serum-free medium containing 10 µm uridine and 10 µm 2′-deoxy-5-fluorouridine that was kept for 3 days to suppress the proliferation of glial cells. Afterward, the medium was changed back to 1 mL BrainPhys Neuron Medium, and half the volume of the medium was replaced twice per week.

For spheroid formation, 1.0 × 10^4^ cells suspended in 150 µL of Neuron Medium were transferred in a well of a 96-well plate (MS-9096 M, Sumitomo Bakelite Co., Ltd.). After centrifugation at 200 × g for 2 min, plates were placed in an incubator at 37 °C and 5% CO_2_. Spheroids were cultured on SCAD devices from day 7. Coated devices equipped with a proprietary seeding jig containing two spheroid mounting holes were immersed into 200 µL of BrainPhys Neuronal Medium in a well of a 24-well plate to prevent devices from drying. Using a 200-µL pipette, a spheroid was transferred into each hole of the jig and settled by centrifugation 300 × g for 3 min. Afterward, half the volume of the medium was replaced twice per week.

### Immunocytochemistry

Sample cultures were fixed with 4% paraformaldehyde in PBS on ice (4 °C) for 10 min. Fixed cells were incubated with 0.2% Triton-X-100 in PBS for 5 min, then with preblock buffer (0.05% Triton-X and 5% FBS in PBS) at 4 °C for 1 h, and finally with preblock buffer containing a specific primary antibody (1:1,000) at 4 °C for 24 h. The primary antibodies used were mouse anti-L glutamate (ab9440, Abcam), rabbit anti-gamma-aminobutyric acid (GABA) (A2052, Sigma-Aldrich), rabbit anti-MAP2 (ab281588, abcom), mouse anti-β-tubulin III (T8578, Sigma-Aldrich), and rabbit anti-MBP (ab40390, Abcam), respectively. Then, the samples were incubated with the appropriate secondary antibody (anti-mouse 488 Alexa Fluor, ab150113, Abcam or anti-rabbit 546 Alexa Fluor, A11010, Lifetechnologies, 1:1,000 in preblock buffer) for 1 h at room temperature. Cell nuclei were counterstained with Cellstain DAPI solution for 1 h at room temperature. Stained cultures were washed twice with preblock buffer (5 min/wash) and rinsed twice with PBS. The immunolabeling was visualized using a confocal microscope (Eclipse Ti2-U, Nikon). Image intensity was adjusted using the ImageJ software (NIH). A Cell3 imager Estier system (Screen Holding) was used to acquire 3D images, and the images were adjusted by a Cell Visualizer software provided by the manufacturer.

### RNA extraction and analysis

Neurons on SCAD device were lysed directly in the culture well by addition of 500 µL TRIzol™ Reagent (15,596,026, Thermo Fisher Scientific). Total RNA was extracted manually using chloroform and isopropyl alcohol solution following manufacturers protocol. An RNA sequencing analysis was entrusted to Agenta Co., Ltd, Tokyo. Briefly, mRNA was extracted by the poly-A selection method targeting mRNA, and the whole genome sequencing was performed using HiSeq X Ten (Illumina Inc.). Expression levels of all mRNAs were presented by the calculated value of fragments per kilobase of exon per million reads mapped (FPKM). After compared the whole FPKM value between hiPSC-derived cortical neurons cultured on SCAD device and those directly cultured on MEA probe for 5 weeks, several typical mRNAs with different expression level (i.e., VGLUT2, GLUR2, NF160, Synaptophysin, Notch1, Nestin, and MASH1) were manually picked-up as shown in Fig. 2C.

### Extracellular recording

Spontaneous extracellular field potentials were acquired at 37 °C under a 5% CO_2_ atmosphere using either a 24-well MEA system (Presto; Alpha Med Scientific) or a CMOS–MEA system (Maxone; Maxwell) at a sampling rate of 20 kHz/channel.

For the Presto system, neurons or neural spheroids cultured on SCAD devices were transferred to MEA plates and incubated for 30 min at 37 °C under 5% CO_2_ atmosphere just before measurements. Signals were high-pass filtered at 1 Hz and stored on a personal computer. The spikes in the acquired data were detected using the 100-Hz high-pass filter.

### Pharmacological tests

Spontaneous activities were recorded for 10 min before treatment and after the cumulative addition to the culture medium of one of the following convulsant agents or receptor antagonists: 4-AP (0.1, 1, 3, 10, or 30 µm; 016–02,781, Wako), pilocarpine (0.1, 1, 3 10, or 30 µm; P6503, Sigma-Aldrich), picrotoxin (0.1, 0.3, 1, 3, or 10 µm; 2,800,471, Nacalai tesque), and AP-5 (1, 3, 10, 30, and 100 µm; 165,304, Sigma-Aldrich). All chemicals were dissolved in DMSO (0.2%–0.6%), which was used as control.

For frequency analysis experiments, 4-AP (0.3, 3, or 30 µm) was administrated into the culture medium, and spontaneous firing was recorded for 10 min.

For propagation analysis experiments using neural spheroids, spontaneous activities were recorded for 10 min before and 20 min after the addition of one of the following typical convulsant agents or receptor antagonists to the culture medium: 4-AP (0.3, 1, 3, 10, or 30 µm), picrotoxin (0.3, 1, 3, 10, or 30 µm), and CNQX (0.3, 1, 3, 10, or 30 µm; C-140, ALOMONE). All chemicals were dissolved in DMSO (0.2%–0.6%), which was used as control.

During all recordings and drug administration, the cultures were kept at 37 °C under a 5% CO_2_ atmosphere.

### Burst analysis

Electrophysiological activity was first analyzed using the Presto software (Alpha Med Scientific) and MATLAB as described before [32] . Briefly, a spike was counted when the extracellularly recorded signal exceeded a threshold of ± 5 σ, where σ was the standard deviation of the baseline noise during quiescent periods. NBs were detected using the 4-step method, which was described previously. Firstly, spikes separated by interspike intervals of 5–15 ms were attributed to the same NB. Secondly, datasets with a maximum number of spikes in the NB below 50–100 spikes/NB were eliminated from the analysis. Thirdly, NBs separated by inter-NB intervals shorter than 100–200 ms were combined. Finally, an NB was defined when it contained more than 500–1,500 spikes/NB. Appropriate numerical values that can accurately detect bursts with 16 electrodes were used as parameter numerical values. All data were expressed as means ± standard errors.

### Frequency analysis

Wavelet analyses were performed using a custom-written program in MATLAB (using function cwt in package “Wavelet Toolbox”) as described before [23] . Briefly, the raw data, *f* (*t*), were transformed as follows:

$$\mathrm{W}\left(\mathrm{b},\mathrm{a}\right)=\frac{1}{\sqrt{a}}{\int }_{-\infty }^{\infty }f\left(t\right)G\left(\frac{t-b}{a}\right)dt$$

where a and b were the scaling factor (1/Hz) and the center location (ms) of the mother wavelet function, respectively, and 1/a varied from 0.1 to 250 Hz. *G*(*x*) is the complex Morlet function:

$$G\left(x\right)=\frac{1}{\sqrt{\pi {F}_{B}}}\mathrm{exp}\left(-\frac{{x}^{2}}{{F}_{B}}\right)\mathrm{exp}\left(2i\uppi {F}_{C}x\right)$$

where *F*_*B*_ = 5 was the frequency bandwidth or wavenumber, and *F*_*C*_ = 1 was the center frequency.

The wavelet power spectrum, W (b, a), is shown. The amplitude of this transform was obtained from its absolute value and color-coded. A scalogram was drawn with the Y-axis representing the frequency band as 181 pixels and the X-axis representing time. One pixel on the X-axis was 50 μs.

$${WT}_{A}=\frac{{WT}_{S}}{{N}_{X} \times {N}_{Y}(f)}$$

*WT*_*A*_: Wavelet transform coefficient per pixel in each frequency band.

*WT*_*S*_: Summation of wavelet transform coefficient in each frequency band.

*N*_*X*_: Number of pixels on X-axis.

*N*_*Y*_(*f)*: Number of pixels on Y-axis, *f* is the frequency band.

### CMOS–MEA measurements

For the Maxone system, neurons cultured on SCAD devices were transferred to CMOS–MEA plates and incubated for 15 min at 37 °C with 5% CO_2_ just before measurement. A whole-sample active scan followed by a local active recording were performed for each sample based on the manufacturer’s protocol. Briefly, about 1,020 electrodes that recorded relative high spike amplitude were selected during a whole-sample active scan divided into several blocks. Then, spontaneous firing activities were recorded on these selected electrodes, and the spike amplitude data were outputted for MATLAB analysis. To calculate the velocity of the synaptic propagation between single firing neurons, the location of firing neurons was determined from the magnitude of the spike amplitude, and a raster plot was generated for the identified neurons to detect the propagation delay between neurons. The network propagation velocity was calculated from the distance between neurons, and the delay before the first spike appeared in the NB of each neuron (Fig. 6A). To calculate axon conduction velocity for peripheral neurons, the pathway map of the axon conduction and the axon traces were identified based on the magnitude of the spike amplitude. Briefly, cell bodies of firing neuron were identified based on the magnitude of the spike amplitude after a local active recording, as described above. Then, the average waveform of every electrode during a very short period before and after the firing time point of one certain identified neuron (i.e., 1.5 ms before the firing time point of cell body and 2.5 ms after that, totally 4 ms), was calculated. And electrodes with relatively similar waveform pattern were manually picked up as the signal pathway of axonal conduction. This progress would be repeated for every identified cell body, to find its pertinent axonal pathway. Finally, the axonal conduction velocity was calculated using a linear fit of the interelectrode distance versus the spike-time latency (Fig. 6B).

### Statistics

One-way ANOVA followed by Dunnett’s test was used to determine the significance of the differences between 2D cultured neurons and neurons cultured on SCAD devices (Fig. 2B), and the differences between each drug concentration and the vehicle (Figs. 3B, 4A, 5C). The differences between between low-frequency components before TBS and after TBS (Fig. 4B) were analyzed using two-tailed paired Student’s t-test.

## Results

### Application of the SCAD device in cell culture and MEA measurements

**Fig. 1:**
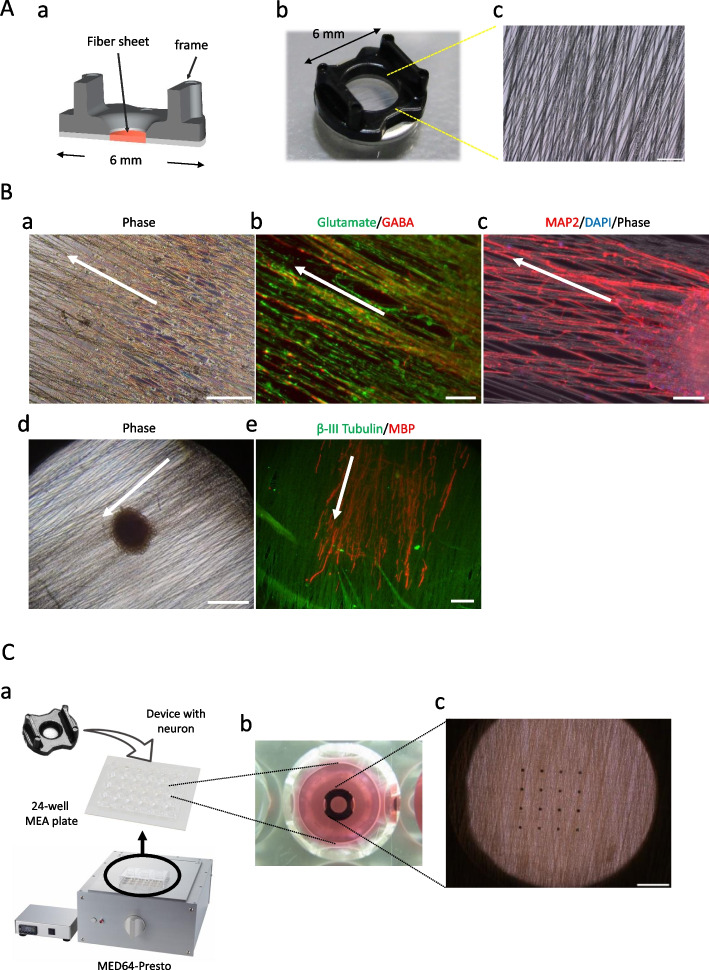
Typical immunostaining showing the features of neuronal cells cultured on SCAD devices. (A) Macroscopic and microscopic images of the SCAD device. (a) Cross-section image. (b) General appearance. (c) Enlarged view of the fiber area. Scale bar = 100 μm. (B) Examples of neuronal cells cultured on SCAD device. (a) Phase contrast image of dorsal root ganglion (DRG) neurons from rat after 5 weeks of in vitro culture (5 WIV). Scale bar = 200 μm. (b) Immunofluorescence images of human induced pluripotent stem cell (hiPSC)-derived glutamatergic (green) and GABAergic (red) neurons at 6 WIV. Scale bar = 100 μm. (c) Immunofluorescence images of hiPSC-derived motor neuron spheroids at 3 WIV stained with an antimicrotubule associated protein 2 (MAP2) antibody. Scale bar = 100 μm. (d) Phase contrast image of spheroid from rat DRGs at 5 WIV. Scale bar = 200 μm. (e) Immunofluorescence images of rat DRGs at 5 WIV. Myelinated axons elongated from the DRG spheroids are stained with anti-βIII Tubulin (green) and antimyelin basic protein (MBP) antibody (red). Scale bar = 100 μm. (C) SCAD device in a multielectrode array (MEA) plate. (a) SCAD device in a Presto MEA system (MED64 Presto, Alpha Med Scientific Inc.). (b) SCAD device in a well of the MEA plate (MED-Q2430L, Alpha Med Scientific Inc.). (c) Enlarged view of the fiber area on the electrodes. Scale bar = 500 μm.

The SCAD device consisted of a stainless-steel washer with polystyrene fibers stretched across the cavity and a polycarbonate frame with handles for easy grasping with tweezers (Fig. 1A-a and b). The outer diameter of the device was 6 mm, which allowed the device to fit into a well of a 96-well plate. The fiber area of the device was a 3-mm diameter circle comprising numerous fibers with a 1–10-μm diameter aligned in one direction or two directions at a 15-degrees angle (Fig. 1A-c).

Figure 1B shows different types of neuronal cells cultured on SCAD devices. When single cells or neuronal spheroids were seeded onto a fiber, axons grew from the cell body along the fibers with the cell body remaining on the fibers. Neuronal axons were entangled with the fibers and were not easily detached, allowing for long-term culture and reliable functional analysis such as immunostainings or electrophysiological measurements by MEA. We confirmed that various types of neurons such as neurons from rat dorsal root ganglia (DRGs) (Fig. 1B-a), hiPSC-derived cortical neurons (Fig. 1B-b), or hiPSC-derived motor neuron spheroids (Fig. 1B-c) could be cultured on the SCAD device. In addition, the presence of the myelin-specific marker myelin basic protein (MBP) was confirmed in cells derived from rat DRGs after 5 weeks of culture on the SCAD device (Fig. 1B-d, e).

Figure 1C shows the device in a well of an MEA plate. The device was easily transferrable from culture plates to MEA plates. By only placing the device on the electrodes in the well, electrophysiological recordings could be performed using MEAs. Figure S2A shows a 3D reconstructed immunofluorescence image of hiPSC-derived cortical neurons cultured on the SCAD device. From the cross section view, neurons have grown through the fiber sheet and reached to the under-layer for over 10 µm after 5 weeks in culture. And the weight of the culture device could significantly influence the measurement result of total number of spikes when transferring to the MEAs (Figure S2B). And the current design of SCAD device could insure enough spike detection from neurons. Taken together, the SCAD device could provide enough neuron contact area and strength to electrodes for electrophysiological measurement after transferring to MEAs.

### Early functional maturation of hiPSC-derived neurons on SCAD devices

**Fig. 2:**
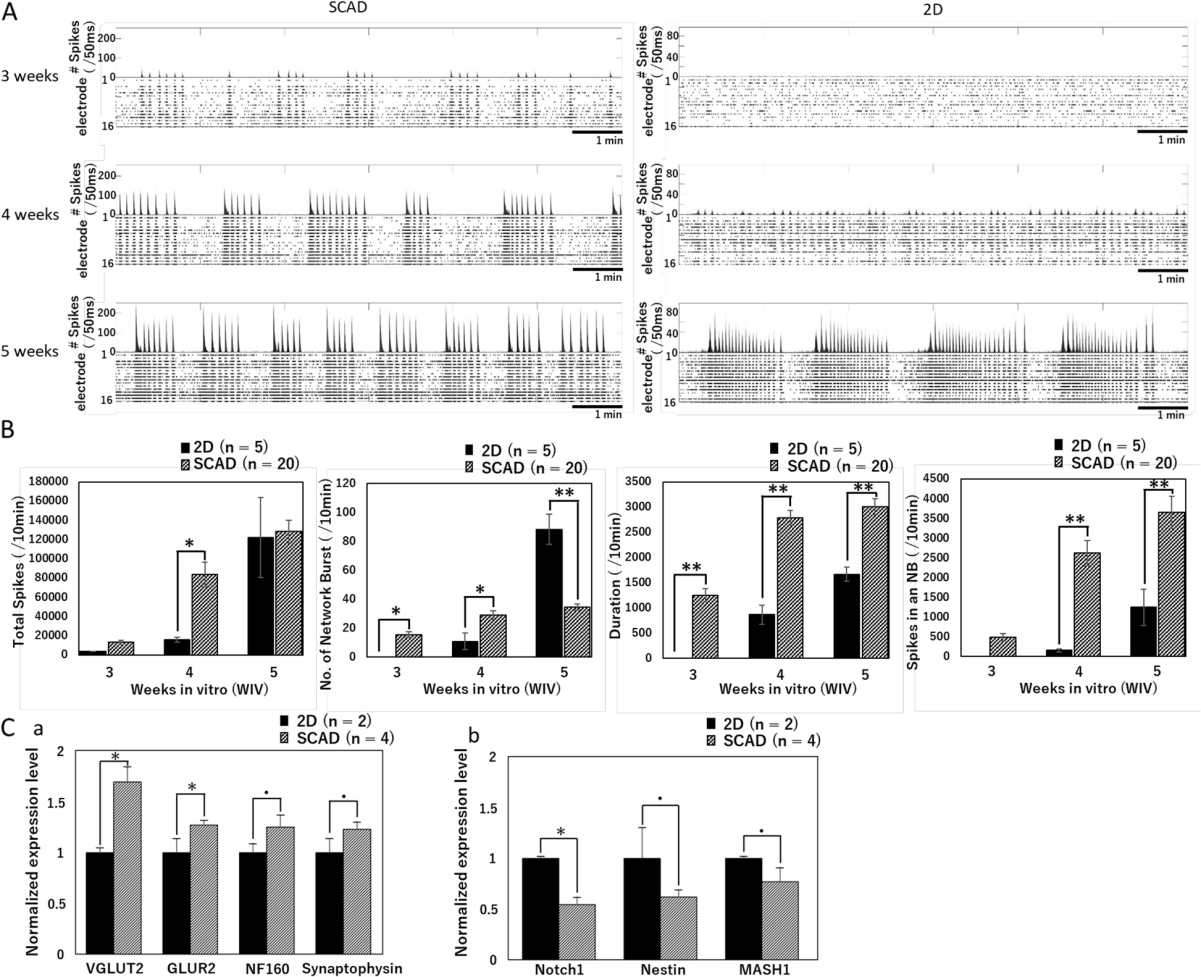
Spontaneous firing of hiPSC-derived cortical neurons cultured on SCAD devices. A) Spike detection rate (number of spikes/50 ms) and corresponding raster plots for all 16 electrodes. Measurements were performed during 10 min at 3, 4, and 5 weeks of in vitro culture (WIV) in neurons cultured on SCAD devices and in 2D cultured neurons. B) Comparison of 4 parameters, i.e., total number of spikes, numbers of network bursts (NBs), duration of NB, and number of spikes in an NB, between 2D cultured samples (*n* = 5) and SCAD cultured samples (*n* = 20) at 3, 4, and 5 WIV. Data were expressed by means + standard errors. Statistical analyses were performed using one-way analysis of variance (ANOVA) followed by post hoc Dunnet’s test, * *p* < 0.05, ** *p* < 0.01. The F value in ANOVA of each parameter is 17.13 for total number of spikes, 40.08 for numbers of NBs, 40.47 for duration of NB, and 16.83 for number of spikes in an NB. C) mRNA levels of typical proteins from 2D cultured neurons (2D, *n* = 2) and neurons cultured on SCAD device (SCAD, *n* = 4). Data were normalized by 2D neurons and expressed by means + standard errors. Statistical analyses were performed using two-tailed paired Student’s t-test, *p < 0.05, *p* < 0.1. a) mRNA levels of VGLUT2 and GLUR2 are significantly higher in SCAD samples than in 2D samples. NF160 and Synaptophysin also expressed higher (*p* < 0.1) in SCAD samples. b) mRNA level of Notch1 is significantly lower in SCAD samples than in 2D samples. Nestin and MASH1 also expressed lower (*p* < 0.1) in SCAD samples.

Functional maturation is important for electrophysiological and pharmacological analyses. To investigate the functional maturation of hiPSC-derived cortical neurons, their spontaneous activity was recorded using a 24-well MEA system (Presto, Alpha Med Scientific Inc.) once every week (Fig. 2). Figure 2A shows representative examples of spontaneous firing patterns, including the spike detection rate and the corresponding raster plots for all 16 electrodes of neurons cultured on SCAD devices or directly on MEA probes (2D culture) for 3, 4, and 5 weeks (3, 4, and 5 weeks in vitro [WIV]). At 3 WIV, network burst (NB) firing was recorded from neurons cultured on the SCAD devices but not from neurons in 2D culture. The number of spikes and NBs was increased in neurons cultured on SCAD devices at 4 WIV compared with that measured at 3 WIV, whereas this was not observed in neurons from 2D cultures. Then, we analyzed 4 parameters related to spike firings, i.e., the total number of spikes, number of NBs, duration of NBs, and number of spikes in an NB, in 20 neuronal samples cultured on SCAD devices and in 5 samples of 2D culture (Fig. 2B). Significantly more total spikes were recorded from SCAD samples than from 2D samples at 4 WIV. At 5 WIV, the total number of spikes was similar between SCAD and 2D samples; however, the variability among samples was smaller in SCAD samples (9.1% Vs 34%). NBs were detected in SCAD samples, but not in 2D samples, already at 3 WIV. At 4 WIV, NBs were observed in 2D samples, but they were fewer than those in SCAD samples, and the variability among samples was high (54%). At 5 WIV, the number of NBs in 2D samples increased significantly, and the variability remained high (12%). In contrast, the number of NBs stayed stable between 4 and 5 WIV in SCAD samples. Additionally, the NB duration was greater at 4 WIV in SCAD samples compared with that in 2D samples, suggesting a better network synchronization in SCAD samples. Since the NB duration did not change at 5 WIV compared with that at 4 WIV in SCAD samples, a stable network synchronization was likely already reached at 4 WIV. The number of spikes in an NB was larger in SCAD samples compared with that in 2D samples at 4 WIV. Although the number of spikes in an NB was increased in 2D samples at 5 WIV, it was still less than that in SCAD samples, suggesting that the network synchronous activity was immature at this time point in 2D samples. Additionally, Fig. 2C shows a comparison result for several typical mRNA levels with different expressions between 2D and SCAD samples at 5 WIV. VGLUT2 and GLUR2, the differential markers for glutamatergic neuron are significantly higher expressed in SCAD samples. And two proteins related to neural maturation, i.e., NF160 as neurofilament protein and Synaptophysin as integral membrane protein are also expressed higher samples. In the other hand, the mRNA levels of Notch1, Nestin, and MASH1, which are proteins that usually expressed in immature neurons but not in mature neurons, are higher in 2D samples than in SCAD samples.

Altogether, these results indicate that the SCAD device promotes a functional network activity in cultured hiPSC-derived cortical neurons at an earlier stage (about 4 WIV) than traditional 2D culture.

### Assessment of the cells’ pharmacological responses using SCAD devices and MEA

**Fig. 3:**
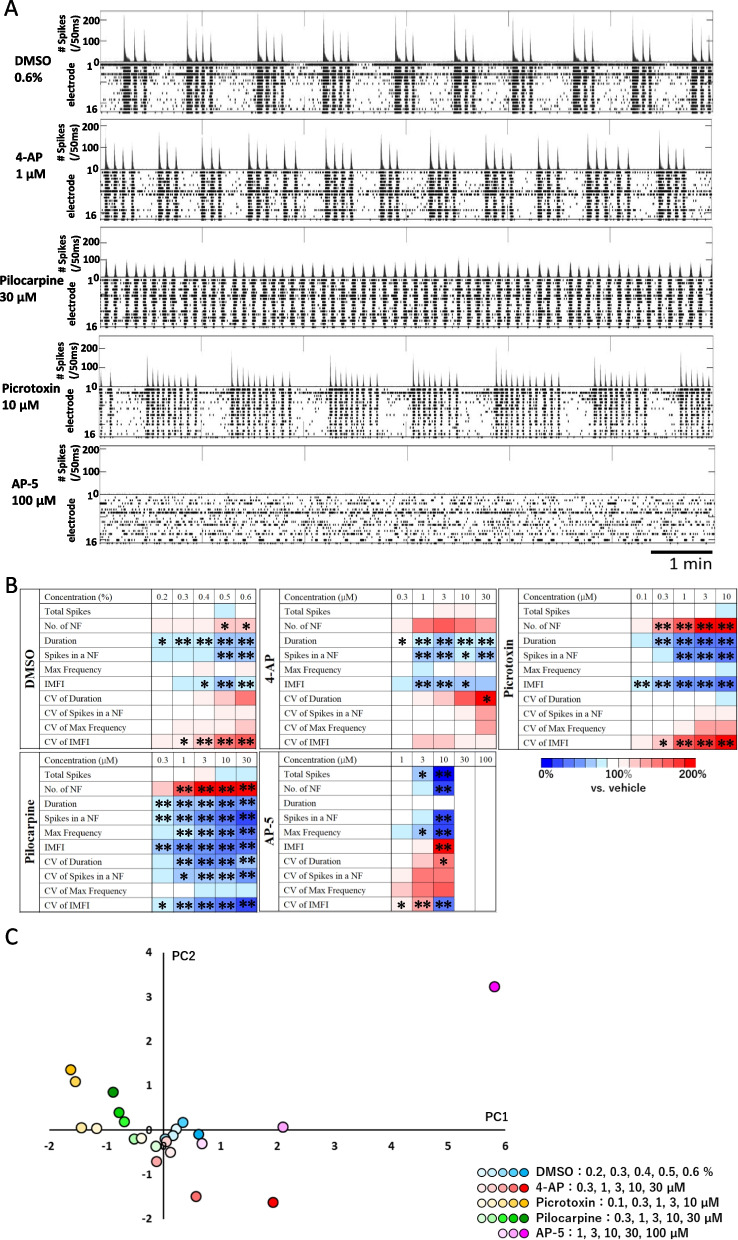
Pharmacological properties of spontaneous firing activity. **A** Typical spontaneous firing patterns of human induced pluripotent stem cell (hiPSC)-derived cortical neurons cultured on SCAD devices after 5 weeks of in vitro culture (5 WIV) after administration of 4-aminopyridine (4-AP), pilocarpine, picrotoxin, and D-( −)-2-amino-5-phosphonopentanoic acid (AP-5) at the indicated concentrations. **B** Heat map of 10 parameters used to analyze the effects of each compound at different concentrations. Statistical analyses were performed using ANOVA followed by post hoc Dunnet’s test, * *p* < 0.05, ** *p* < 0.01. **C** Scatter plots of principal component analysis (PCA) using a 5-parameter set (total number of spikes, duration of network bursts (NBs), intermaximum frequency interval (IMFI), coefficient of variation (CV) of NB duration, and CV of the number of spikes in an NB) for detecting drug effects

To evaluate the efficacy of drug treatments of hiPSC-derived cortical neurons cultured on SCAD devices, we measured changes in the spontaneous activity following the cumulative administration of three typical convulsant agents, i.e., 4-Aminopyridine (4-AP), pilocarpine, and picrotoxin, and an antagonist of the N-methyl-D-aspartate (NMDA) glutamate receptor D-(–)2-amino-5-phosphonopentanoic acid (AP-5) at 5 WIV (Fig. 3 and Supplementary Fig. 1). Representative changes of spike detection rate and raster plots obtained for each compound are shown in Fig. 3A, and detailed changes in response to every concentration of each compound are shown in Figure S1. Compared with neurons receiving vehicle (dimethyl sulfoxide, DMSO), a concentration-dependent increase of the number of NBs was observed in neurons treated with pilocarpine or picrotoxin. AP-5 induced a concentration-dependent decrease in the number of NBs and even a disappearance of NBs for an AP-5 concentration of 30 µm. To identify analytical parameters accurately detecting drug efficacy, we generated a heat map of 10 analytical parameters after administration of the compounds (one-way analysis of variance [ANOVA] followed by Dunnett’s test, Fig. 3B). After administration of 4-AP, pilocarpine, and picrotoxin, the number of NBs increased in a concentration-dependent manner. The total number of spikes and the number of NBs decreased with increasing concentrations of AP-5, and no network synchronous activity was detected in cells treated with 30 µm AP-5. The coefficient of variation (CV) of the NB duration increased with the concentration of 4-AP. The CV of intermaximum frequency interval (IMFI) increased with the concentration of picrotoxin. All CVs (i.e., CV of the duration of an NB, CV of the number of spikes in an NB, CV of the maximum frequency, and CV of IMFI) were decreased with increasing concentrations of pilocarpine. The results of a principal component analysis (PCA) using a five-parameter set (total number of spikes, duration of an NB, IMFI, CV of duration of an NB, and CV of the number of spikes in an NB) are shown in Fig. 3C. We compared the first 2 principal components obtained after administration of the vehicle DMSO and 4 compounds. The concentration of DMSO demonstrated no effect (one-way multivariate ANOVA [MANOVA], Table 1). Conversely, the four components demonstrated significantly different effects than those of DMSO, and the effects of the four compounds were different from each other (Table 2). These results suggested that the pharmacological responses of hiPSC-derived cortical neurons cultured on SCAD devices were as expected as they translated the drugs’ mechanisms of action.

**Table 1:** Statistics of the principal component analysis (PCA) of different dimethyl sulfoxide (DMSO) concentrations

DMSO concentration (v/v)	*p* value				
	vs 0.20%(v/v) DMSO	vs 0.30%(v/v) DMSO	vs 0.40%(v/v) DMSO	vs 0.50%(v/v) DMSO	vs 0.60%(v/v) DMSO
0.20%		*p* = 0.92	*p* = 0.83	*p* = 0.85	*p* = 0.87
0.30%	*p* = 0.92		*p* = 0.96	*p* = 0.65	*p* = 0.90
0.40%	*p* = 0.83	*p* = 0.96		*p* = 0.61	*p* = 0.86
0.50%	*p* = 0.85	*p* = 0.65	*p* = 0.61		*p* = 0.72
0.60%	*p* = 0.87	*p* = 0.90	*p* = 0.86	*p* = 0.72	

*N* = 4 for each concentration (0.2%, 0.3%, 0.4%, 0.5%, and 0.6%) of DMSO. Statistical analyses were performed using one-way analysis of variance (ANOVA) followed by post hoc Dunnet’s test

**Table 2:** Statistics of the principal component analysis (PCA) of different compounds

Compound	*p* value				
	vs DMSO	vs 4-AP	vs Picrotoxin	vs Pilocarpine	vs AP-5
DMSO		* p < 0.01	* p < 0.01	* p < 0.01	* p < 0.01
4-AP	* p < 0.01		* p < 0.01	* p < 0.01	* p < 0.01
Picrotoxin	* p < 0.01	* p < 0.01		* p < 0.01	* p < 0.01
Pilocarpine	* p < 0.01	* p < 0.01	* p < 0.01		* p < 0.01
AP-5	* p < 0.01	* p < 0.01	* p < 0.01	* p < 0.01	

*N* = 4 for each compound (dimethyl sulfoxide [DMSO], 4-aminopyridine [4-AP], picrotoxin, pilocarpine, and D-( −)-2-amino-5-phosphonopentanoic acid [AP-5]). Statistical analyses were performed using ANOVA followed by post hoc Dunnet’s test, *p < 0.01

### Analysis of the frequency of spontaneous activity triggered by compound administration and of evoked activity after electrical stimulation

**Fig. 4:**
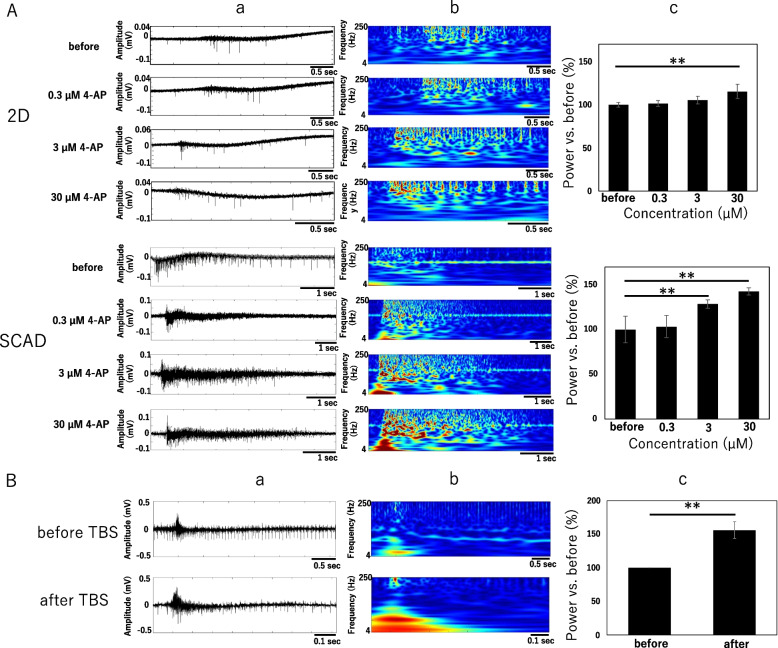
Analysis of the signal low-frequency components after compound administration or electrical stimulation. A) Frequency analysis of the signal from 2D cultured neurons and neurons cultured on SCAD devices treated with 4-aminopyridine (4-AP) administration (0.3, 3, and 30 µm). a) Local field potential (LFP) of a network burst (NB) before and after administration of 0.3, 3, and 30 µm 4-AP recorded with the same electrode. LFPs high-pass filtered at 1 Hz were recorded. b) Corresponding scalograms of the LFPs measured during the application of 4-AP. The scalograms are computed from the raw traces, not the high-pass filtered data. c) Changes in the signal low-frequency component (below 250 Hz) after 4-AP administration normalized to those of controls measured in 2D cultured neurons and neurons cultured on SCAD devices. Data were quantified using the average wavelet transform coefficient per pixel in the corresponding scalogram, *n* = 6 for 2D cultured neurons and *n* = 8 for neurons cultured on SCAD devices. Data were expressed by means ± standard errors. Statistical analyses were performed using ANOVA followed by post hoc Dunnet’s test, ** *p* < 0.01. B) Frequency analysis of neurons cultured on SCAD devices before and after theta burst stimulation (TBS). a) LFPs of an NB before and after TBS obtained using the same electrode. b) Scalograms of the LFPs measured before and after TBS. c) Changes in the signal low-frequency component (below 250 Hz) before and after TBS. Data were quantified using the average wavelet transform coefficient per pixel in the corresponding scalogram, *n* = 5. Data were expressed by means ± standard errors. Statistical analyses were performed using two-tailed paired Student’s t-test, ** *p* < 0.01

New parameters need to be defined to evaluate in vitro the functions of neural networks. Using neurons cultured on SCAD devices, we focused on the analysis of low-frequency components of NBs as a parameter to detect the pharmacological response of spontaneous activities and the evoked response after electrical stimulation. Figure 4A-a and -b show a typical local field potential and the wavelet analysis of NBs before and after treatment with 0.3, 3, and 30 µm 4-AP of 2D cultured neurons or neurons cultured on SCAD devices. A wavelet analysis of components with frequencies below 250 Hz was performed. From the scalograms obtained after wavelet analysis (Fig. 4A-b), a concentration-dependent increase of low-frequency components (below 250 Hz) was found in cells cultured on SCAD device and in 2D after 4-AP treatment. Quantification was performed by calculating the wavelet coefficient per pixel of the scalogram. It showed that the power of the low-frequency component signal increased by 115.3% in 2D cultured samples and 142.5% in SCAD cultured samples treated with 30 µm 4-AP (Fig. 4A-c).

To evaluate neural network functions including synaptic transmission efficiency, theta burst stimulation (TBS) was applied to neurons cultured on SCAD devices, and the low-frequency components (below 250 Hz) were analyzed before and after TBS as described above (Fig. 4B). The scalogram obtained after wavelet analysis (Fig. 4B-b) showed that more low-frequency components were observed after TBS. The quantitative analysis revealed that the power of the low-frequency components increased by 156.1% after TBS compared with that before TBS (Fig. 4B-c). Therefore, analyzing low-frequency components of NBs is an effective index for evaluating synaptic transmission efficiency in neurons cultured on SCAD devices.

### Analysis of the signal propagation velocity analysis neural spheroids cultured on SCAD devices

**Fig. 5:**
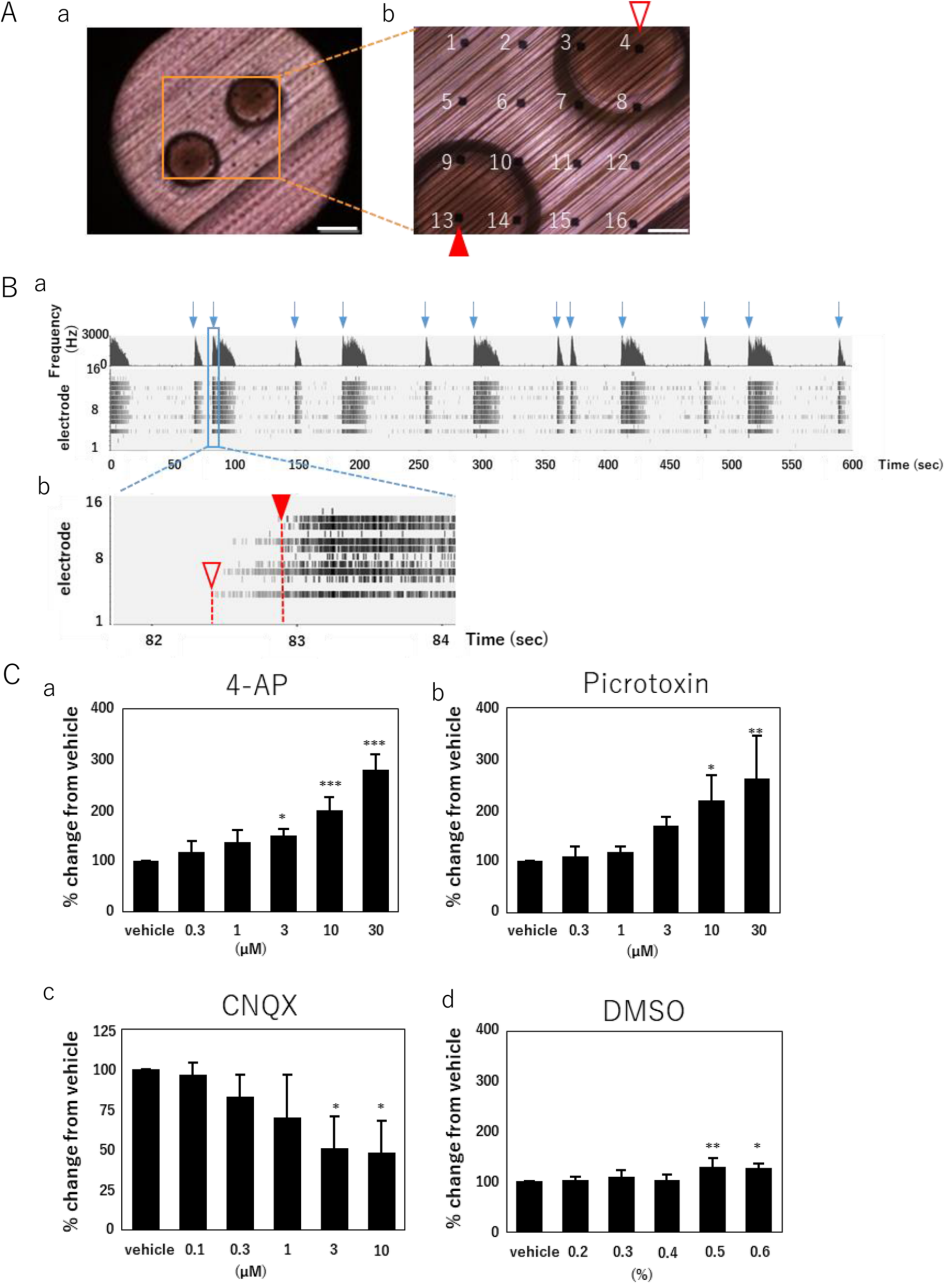
Propagation velocity of the signal between neuronal spheroids cultured on SCAD devices. (A) (a) Two spheroids on the seeding jig in the SCAD device. Scale bar = 500 μm. (b) Enlarged view of the spheroids. The numbers indicate the electrode number in a well of a multielectrode array (MEA) plate. Blank and filled arrowheads indicate the electrodes (#4 and #13, respectively) used for propagation velocity analysis. Scale bar = 100 μm. B) Representative histogram and raster plot acquired for the spheroid sample cultured 5 weeks in vitro (at 5 WIV). (a) The vertical axes represent the spike frequency (upper) and electrode numbers in a well of an MEA plate (lower). The horizontal axis represents the measurement duration. Arrows indicate the bursts analyzed at the time of firing onset. Blank and filled arrowheads corresponded to those shown in A and indicate the beginning point of the NB detected at each electrode. C) Dose-dependency effects of drugs on the propagation velocity in spheroid samples at 5 WIV. More than 30 bursts recorded for 10 min were analyzed for each sample. For 4-aminopyridine (4-AP) *n* = 4, for picrotoxin *n* = 3, for 6-cyano-7-nitroquinoxaline-2, 3-dione (CNQX) *n* = 3, for dimethyl sulfoxide (DMSO) *n* = 4. Data were expressed by means + standard errors. Statistical analyses were performed using ANOVA followed by post hoc Dunnet’s test, *p < 0.05, **p < 0.01, ***p < 0.001

Neurons cultured on SCAD devices formed a neural network with their axons intimately connected. By measuring the potentials generated from different cells using individual electrodes, measuring the excitation delay in different cells (i.e., propagation velocity) was possible. For analysis with the MEA system, two spheroids were mounted on a SCAD device consisting of unidirectional fibers (Fig. 5A-a). Each spheroid was placed on the diagonal electrodes (Fig. 5A-b), and after a few weeks of culture, a neural network formed between the spheroids, which extended axons toward each other. After 2 weeks of spheroid formation and 3 weeks of culture on the device, neuronal firing and NBs were detected (Fig. 5B-a and b). When comparing the time at which a particular NB began, a slightly delayed onset of firing (375 ± 166 ms, mean ± SD, *n* = 31) was measured with the electrodes 4 and 13, on top of which the spheroids were sitting (Fig. 5B-b). The propagation velocity was calculated using the delay duration, and the distance between the electrodes and was 3.73 mm s^−1^. The treatment with 4-AP or picrotoxin increased the relative velocity in a dose-dependent manner compared with that measured in vehicle-treated samples, suggesting an acceleration of the neuronal activity in response to the drugs (Fig. 5C-a and b). In samples treated with 3 and 10 μM 6-cyano-7-nitroquinoxaline-2, 3-dione (CNQX), an antagonist of the alpha-amino-3-hydroxy-5-methyl-4-isoxazolepropionic acid (AMPA) and kainate glutamatergic receptors, the relative velocity was significantly reduced compared with that of controls (Fig. 5C-c). In DMSO-treated samples, the relative velocity change was significantly, albeit slightly, increased by 0.5% and 0.6% DMSO. These results suggest that the detection of delayed propagation allowed to discriminate between mutually activated neurons and the alterations of the propagation velocity reflected the drug’s mode of action.

### Measurement of network propagation velocity and axonal conduction velocity using complementary metal-oxide semiconductor (CMOS)–MEA

**Fig. 6:**
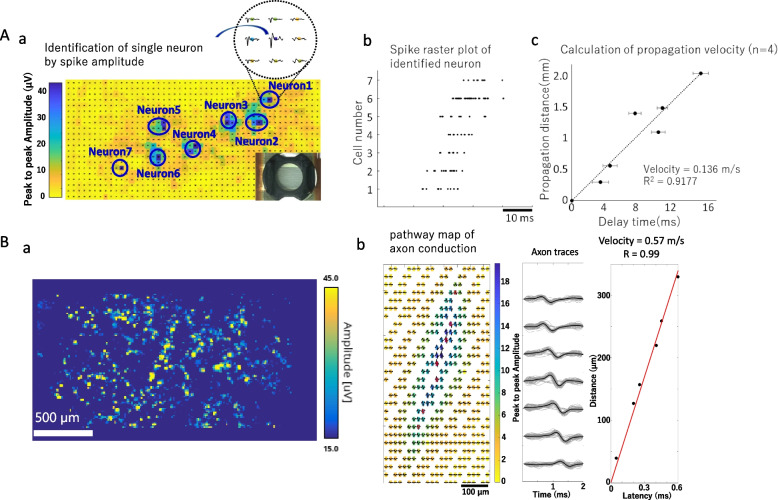
Network signal propagation velocity in hiPSC-derived cortical neurons and axonal conduction velocity in DRG neurons. A) Network propagation velocity in hiPSC-derived cortical neurons cultured on SCAD devices. a) Whole-sample spike amplitude map obtained with complementary metal-oxide semiconductor–multielectrode array (CMOS–MEA). Seven neurons were identified using the magnitude of spike amplitude. b) Spike raster plot for each neuron identified in a). c) Signal propagation velocity between neurons from the network. It was calculated from the distance between neurons and the delay between the first spike of a network burst of each neuron, *n* = 4. B) Measurement of axonal conduction velocity of dorsal root ganglion (DRG) neurons cultured on SCAD devices. a) Whole-sample spike amplitude map. b) Example of the calculation of axonal conduction velocity for one single neuron. A pathway map, extracted axon traces, and a linear fit were used to calculate the velocity

Action potential propagation was detected through a synaptic network formed by two neural spheroids connected through their axons extended along aligned fibers of the SCAD device. However, neurites of hiPSC-derived cortical neurons cultured on SCAD devices were more intricately developed than those of spheroids, and these cells’ firing patterns were more complex. Therefore, the network propagation velocity was difficult to assess using a traditional 16-electrodes MEA. To solve this problem, we established a method for measuring network propagation velocity in hiPSC-derived cortical neurons cultured on SCAD devices using a 26,400-electrodes CMOS–MEA (Maxwell Biosystems, inc.). Since CMOS–MEA provides a high spatial resolution, the signal from one single neuron can be measured at multiple electrode points, allowing to determine the location of the cell body from the magnitude of the spike amplitude. We identified the locations of 7 firing neurons using the magnitude of the spike amplitude and then generated a raster plot for the identified neurons. The plots revealed a clear propagation delay among the 7 neurons (Fig. 6A). Since this propagation delay was detected repeatedly, the network propagation velocity between neurons was calculated using the distance between neurons and the delay before recording the first spike of an NB in each neuron (Fig. 6A). The network propagation velocity was 0.14 m s^−1^ (R^2^ = 0.92, *n* = 4), which is a plausible value for synaptic propagation.

Since single firing neurons could be identified using CMOS–MEA, we attempted to measure axonal conduction velocity for peripheral neurons cultured on SCAD devices. Dissociated DRG neurons isolated from 10-week-old rats were cultured on SCAD devices for 4 weeks, and then, their spontaneous activity was measured using CMOS–MEA. Following whole-sample activity imaging, we identified the location of firing neurons using the spike amplitude (Fig. 6B a). Then, a local recording using only electrodes around one single neuron was performed. We generated the pathway map of axonal conduction using the magnitude of the average spike amplitude and identified axon traces (Fig. 6B b). The axonal conduction velocity was 0.57 m s^−1^ (R^2^ = 0.99), a plausible value for unmyelinated nerves.

## Discussion

The SCAD device introduced in the present study was convenient to utilize for neuron functional analysis using MEAs as it was easily transferred to different MEA plates, which might contribute to decrease the experimental expenses. We demonstrated the potential of SCAD device for early functional maturation of hiPSC-derived neural networks. We also measured the spontaneous activities induced by clinical convulsants to demonstrate the efficiency of the culture model for analyzing pathological mechanisms. In addition, low-frequency components were analyzed to investigate in vitro neural network functions. We showed that the synaptic propagation velocity was detected by MEA in neurons cultured on SCAD devices. These parameters allowed to predict neural network activities during drug testing.

An increasing number of cell culture models use biomaterial scaffolds to provide an adequate surface for cell adhesion and to enable efficient cell proliferation, differentiation, and organization into a mature and functional engineered tissue [33–35] . Several synthetic polymers such as poly- + -caprolactone (PCL), polylactic acid, and polyglycolic acid have been utilized as biomaterial scaffolds in cell culture to generate three-dimensional (3D) tissues. In neuronal cell culture, the oriented fibers obtained with these polymers contribute to the parallel axon guidance, thus mimicking the native tissue environment [36–38]. Considerably, a neuron culture system based on such polymers should also be welcomed for evaluating neuronal electrophysiological properties in need of drug screening or neurotoxicity testing. In the present study, the SCAD device constituted of ESPS fibers shows potential as a finely structured substrate for long-term neuron culture preserving the axon orientation and preventing cell detachment or aggregation. Neurons cultured on SCAD devices were aligned along individual fibers, potentially because of the alignment of polymer chains within the larger microfibers [39] . The cells could be seeded and cultured as single cells or spheroids on the SCAD device. Axon outgrowth along the fiber was promoted, and axons were entangled with the fiber, allowing for sample immunostaining without sample loss due to cell detachment or aggregation. From immunostaining images, in vitro myelination is also confirmed in rodent peripheral neurons after a long-term culture on SCAD device. Overall, the SCAD device might provide a fine scaffold for the long-term culture of either hiPSC-derived neurons or rodent primary neurons with oriented neurite development. The main advantage to using SCAD devices is that it fitted in 96-well plates and was convenient to transfer from the cell culture plates to the MEA probes, which allowed to rapidly and repeatedly characterize the neuron electrophysiological properties. As we shown here, SCAD devices are currently compatible with both MED64-Presto (Alpha Med Scientific Inc.) and Maestro series (Axion BioSystems, Inc.) MEA systems, and a CMOS-MEA system (Maxwell Biosystems, inc.).

In hiPSC-derived neurons, spontaneous NB firing is important for transferring information within the cortex and is an indicator of functional maturation [21–24]. As shown in Fig. 2, NBs were detected in hiPSC-derived cortical neurons cultured on SCAD devices at 3 WIV, which is earlier than NBs are measured in neurons cultured on MEA probes. Additionally, neurons cultured on SCAD devices show constant NB firing at 4 and 5 WIV, indicating that neurons achieved functional maturation earlier than neurons in traditional 2D culture. In 2D culture, neurites are considered to be randomly orientated, whereas they developed along the aligned fibers of the SCAD device. Therefore, signal transduction was more efficient due to the unidirectional network formation, and one single neuron could activate neighbor neurons contacted through its axon with a strong synaptic input, resulting in an early apparition of NBs. Leong’s group also reported that ESPS scaffolds accelerate the differentiation of hiPSC-derived cells to a bioreactor model in culture with minimal external manipulation, which agrees with the present study [40]. Finally, in conventional MEAs, neurons need to be cultured for over 6 weeks before drug testing [24]. Therefore, the early functional maturation achieved with the SCAD device allows for more efficient electrophysiological analyses.

Predictable and stable responses are important for the standardization of drug screenings using the MEA system. After 5 weeks of culture, the administration of AP-5 strongly decreased, or even abolished at higher concentrations, the number of spikes and NBs. These data suggested that hiPSC-derived cortical neurons cultured on SCAD devices expressed mature NMDA glutamatergic receptors at 5 WIV. The NMDA receptor is important for neuroplasticity involved in learning, and excess of NMDA is implicated in numerous acute and chronic neurodegenerative diseases [41, 42]. After the administration of pilocarpine, the number of NBs was increased while all CV values were decreased. This result indicated that pilocarpine could induce a prominently periodicity in neural spontaneous firing, which agreed with a previous research by our group [30]. Additionally, the administration of 4-AP, pilocarpine, and picrotoxin induced an increasing number of NBs in a dose-dependent manner, which agrees with previous reports on cells’ response to these typical convulsant agents. For example, concentrations above 10 µm of 4-AP induced a significant increase in the number of NBs, and previous in vivo studies reported that 4-AP blood levels above 10 µm provoke seizures in animal models [43, 44]. Similar effects were observed for pilocarpine (above 1 µm) and picrotoxin (above 1 µm) [45–48]. Thus, in vivo phenomenons could be reproduced in neurons cultured on SCAD device. Altogether, our data suggest that neurons cultured on SCAD devices are suitable for drug screening.

To investigate in vivo neural network functions more efficiently with MEAs, parameters of synaptic activity need to be defined. Here we analyzed the low-frequency, below 250 Hz, band of NB in neurons cultured on SCAD devices. The amount of low-frequency components was increased by 4-AP administration in a dose-dependent manner. This increase was more significant in neurons cultured on SCAD devices than that observed in 2D cultured neurons, possibly because neurons on SCAD devices formed unidirectional networks. Indeed, the oriented axonal network might facilitate signal propagation through the synaptic network. Considering that high-frequency β and γ waves are enhanced during epilepsy seizures in humans [49–51], in vitro frequency analysis of cultured neural networks using MEA is a useful tool. Furthermore, we showed that TBS induced a significant increase of the low-frequency components in neurons cultured on SCAD devices. In acute brain slices, TBS is commonly used to induce long-term potentiation (LTP) [52], and it also leads to an augmentation of the low-frequency components. Therefore, frequency analysis of neurons cultured on SCAD devices might be used to predict LTP-relevant neural network functions.

Analyzing the velocity of the signal propagating through multiple synapses is another approach for evaluating neural network functions [53]. Here, we successfully measured the signal propagation velocity between two neural spheroids and between single firing neurons cultured on SCAD devices by detecting the delayed excitation. Drug treatment at different concentrations of neural spheroids affected the signal propagation velocity, indicating that the propagation velocity might be a useful parameter to investigate a drug effect on neural network functions. Also, we successfully calculated the signal propagation velocity in cultured hiPSC-derived cortical neurons using a CMOS–MEA system. Using similar drug administration experiments and analysis methods to those used for spheroids, we were able to predict drug efficacy.

Changes in axon conduction velocity are commonly used for clinical diagnosis of peripheral neuropathy. Previous in vitro studies direct axonal alignment in polydimethylsiloxane models and show conduction velocity changes under drug administration [54, 55]. The SCAD device also allowed the unidirectional alignment of axons and, consequently, enabled to reproduce the axon conduction velocity of DRG neurons in culture on SCAD devices as measured using the CMOS–MEA system. The axonal conduction velocity we got here was closer to that collected in animal experiments [56] compared to previous reports, which could be due to high spatiotemporal resolution provided by the CMOS-MEA system [53]. Therefore, measuring axon conduction velocity in neurons cultured on SCAD devices is likely suitable for evaluating peripheral toxicity.

## Conclusions

In the present study, we showed that the SCAD device is a fine in vitro culture system for both hiPSC-derived neurons and primary peripheral neurons. A notable advantage of the SCAD device is convenient application for MEA analysis, which could contribute to decrease related experimental costs. We showed that early functional maturation of hiPSC-derived neurons could be confirmed by culturing on SCAD device. Additionally, after maturation, neurons responded to convulsant agents as expected, suggesting that this system can be used to evaluate drugs’ efficacy. Furthermore, we successfully identified new parameters, i.e., low-frequency components and synaptic propagation velocity, reflecting neural network functional changes induced by drug administration. Finally, we developed a method to measure the axon conduction velocity of primary peripheral neurons cultured on SCAD devices. Thus, MEA of neurons cultured on SCAD devices is proposed as a reliable in vitro platform for evaluating neuron functions, drug efficacy or toxicity.

SCAD devices might contribute greatly to elucidating the mechanisms of neuropathologies.

## Supplementary Information

**Additional file 1:** **Figure 1.** Typical spontaneous firing patterns of hiPSC-derived cortical neurons cultured on SCAD devices for 5 WIV after administration of different concentrations of vehicle or various compounds.

**Additional file 2:** **Figure 2.** A) A 3D reconstructed immunofluorescence image of hiPSC-derived cortical neuron cultured on the SCAD device. Pictures are acquired by Cell3 imager Estier, and reconstructed by Cell Visualizer (Screen Holding Co., Ltd).

## Data Availability

The data that support the findings of this study are available from the corresponding author upon reasonable request.

## Abbreviations

MEA: Microelectrode array
hiPSC: Human induced pluripotent stem cells
ESPS: Electrospun polystyrene
DRG: Dorsal root ganglia
WIV: Weeks in vitro
NB: Network burst
TBS: Theta burst stimulation
