# Supplementary material for: A functional neuron maturation device provides convenient application on microelectrode array for neural network measurement
Source: Biomater Res. 2022 Dec 20;26:84. doi: 10.1186/s40824-022-00324-z (PMC9768978; doi:10.1186/s40824-022-00324-z)
Supplement: Supplementary file 1 — Additional file 1: Figure 1. Typical spontaneous firing patterns of hiPSC-derived cortical neurons cultured on SCAD devices for 5 WIV after administration of different concentrations of vehicle or various compounds. [file 40824_2022_324_MOESM1_ESM.pdf]

Additional file 1

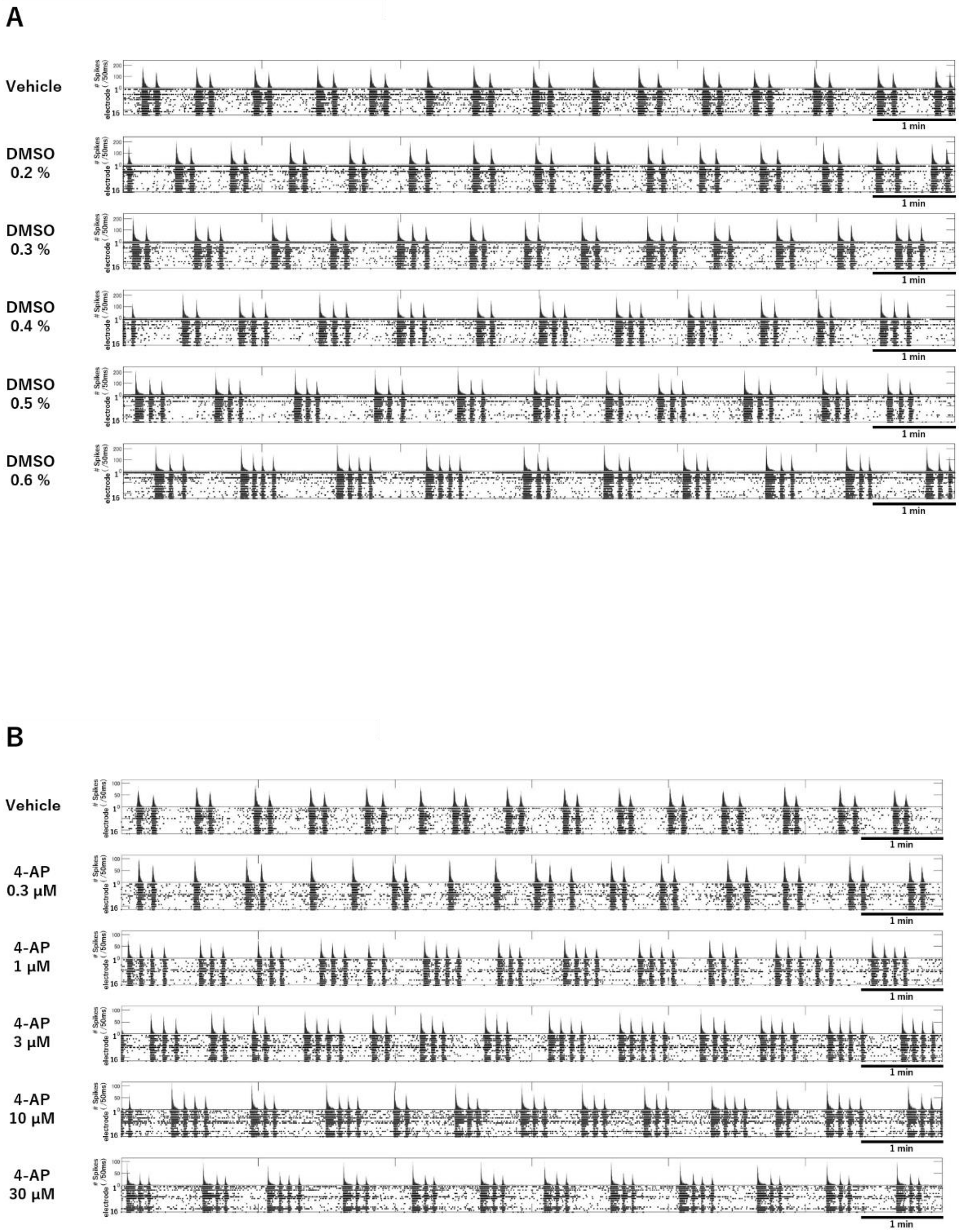

C

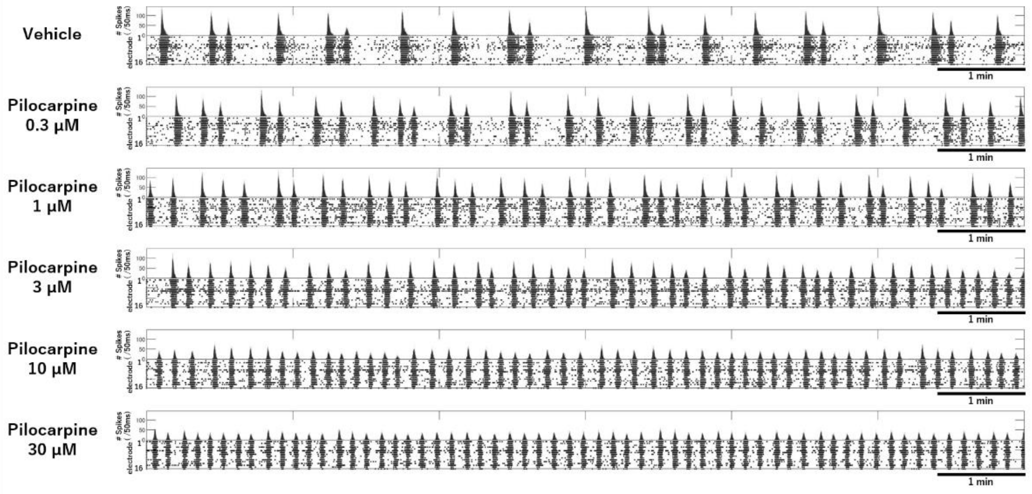
Vehicle
Pilocarpine 0.3 μM
Pilocarpine 1 μM
Pilocarpine 3 μM
Pilocarpine 10 μM
Pilocarpine 30 μM
Spikes (/50ms)
electrode
1 min

D

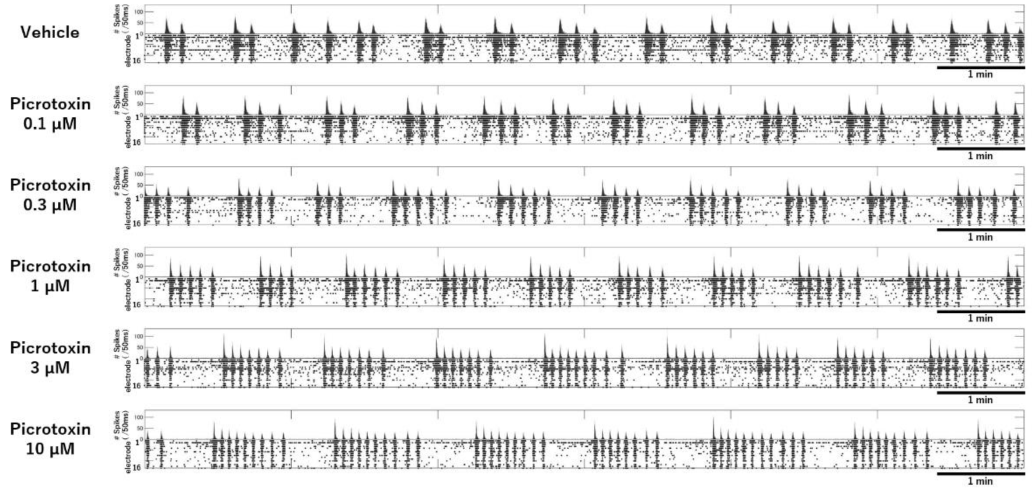
Vehicle
Picrotoxin 0.1 μM
Picrotoxin 0.3 μM
Picrotoxin 1 μM
Picrotoxin 3 μM
Picrotoxin 10 μM
Spikes (/50ms)
electrode
1 min

E

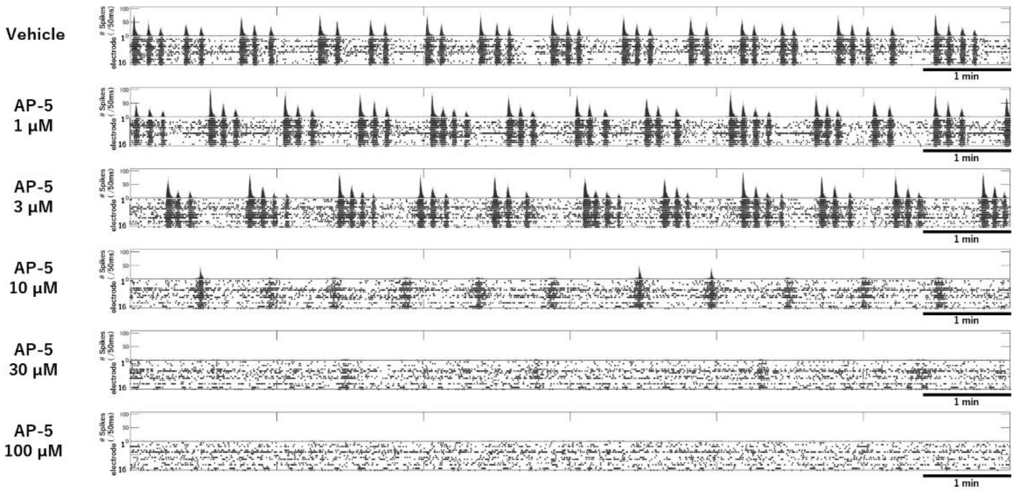

Supplementary Figure 1: Typical spontaneous firing patterns of human induced pluripotent stem cell (hiPSC)-derived cortical neurons cultured on SCAD devices for 5 weeks (5 WIV) after administration of different concentrations of vehicle (dimethyl sulfoxide [DMSO]) or various compounds.

A) DMSO at 0.2%, 0.3%, 0.4%, 0.5%, and 0.6%.

B) 4-aminopyridine (4-AP) at 0.3, 1, 3, 10, and 30 μM.

C) Pilocarpine at 0.3, 1, 3, 10, and 30 μM.

D) Picrotoxin at 0.1, 0.3, 1, 3, and 10 μM.

E) D-(−)-2-amino-5-phosphonopentanoic acid (AP-5) at 1, 3, 10, 30, and 100 μM.
