# Supplementary material for: A functional neuron maturation device provides convenient application on microelectrode array for neural network measurement
Source: Biomater Res. 2022 Dec 20;26:84. doi: 10.1186/s40824-022-00324-z (PMC9768978; doi:10.1186/s40824-022-00324-z)
Supplement: Supplementary file 2 — Additional file 2: Figure 2. A) A 3D reconstructed immunofluorescence image of hiPSC-derived cortical neuron cultured on the SCAD device. Pictures are acquired by Cell3 imager Estier, and reconstructed by Cell Visualizer (Screen Holding Co., Ltd). [file 40824_2022_324_MOESM2_ESM.pdf]

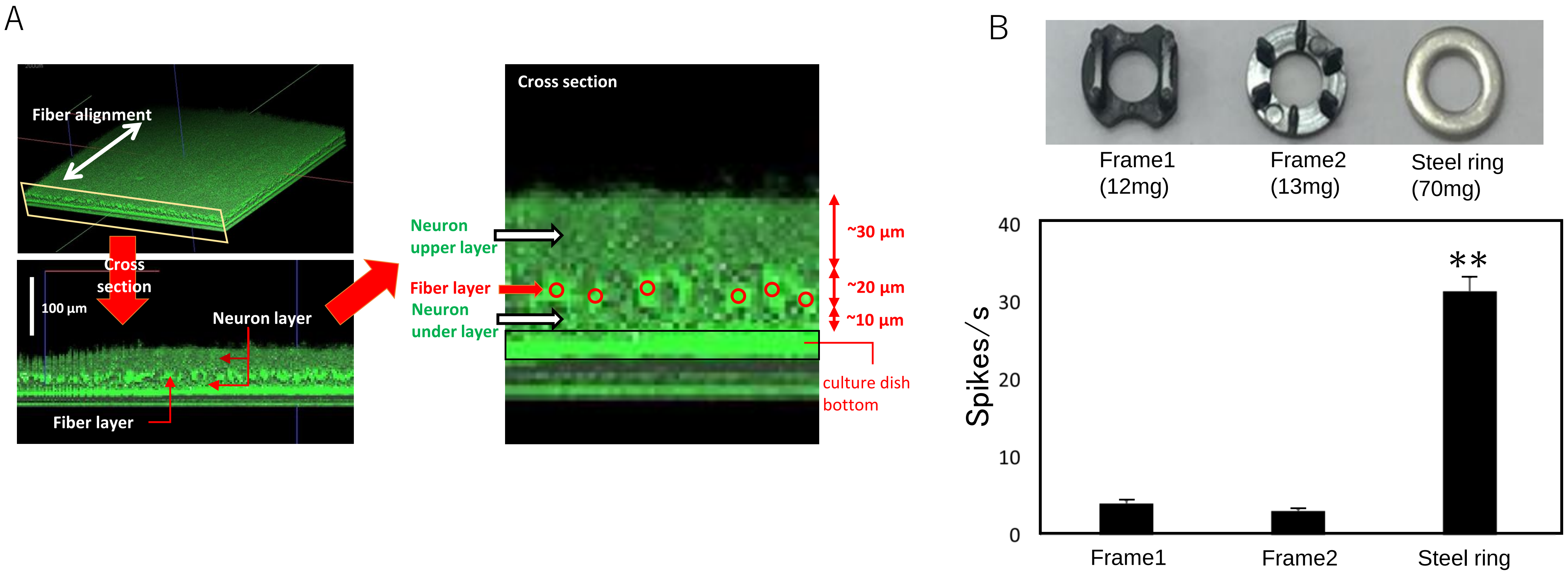

Supplementary Figure 2.

A) A 3D reconstructed immunofluorescence image of hiPSC-derived cortical neuron cultured on the SCAD device. Pictures are acquired by Cell3 imager Estier, and reconstructed by Cell Visualizer (Screen Holding Co., Ltd).

B) Comparison of total number of spikes from neurons cultured on devices with three different weights. Measurements were performed during 15 min at 3 WIV in cultured primary rodent cortical neurons. Data were expressed by means + standard errors, n=6. Statistical analyses were performed using one-way analysis of variance (ANOVA) followed by post hoc Dunnet’s test, ** $p < 0.01$.
